# Development of the mammary glands and its regulation: how not all species are equal

**DOI:** 10.1093/af/vfad029

**Published:** 2023-06-14

**Authors:** Adam J Geiger, Russell C Hovey

**Affiliations:** Zinpro Corporation, Eden Prairie, MN 55344, USA; Department of Animal Science, University of California, Davis, CA 95616, USA

**Keywords:** endocrinology, epithelium, morphogenesis, nutrition, stroma

**Implications**

- Species-specific development of the mammary glands (MG) necessitates the use of appropriate animal models in research.
- The MG of livestock represent a unique model for modeling human breast development.
- The complexities of MG development reveal unique growth regulatory mechanisms.
- The MGs are responsive to nutritional intervention that increases their growth and lactational performance.

## Introduction

**Figure 1.**
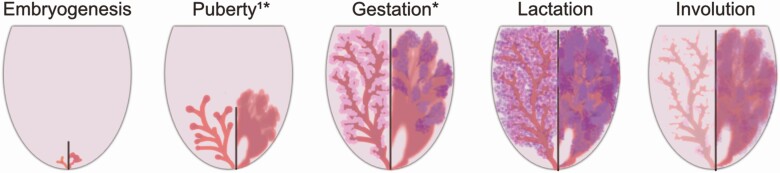
Diagrammatic representation of the stages of mammary gland development in the female mouse (left half of each panel) or bovine (right half of each panel) mammary gland. *Puberty and gestational development are associated with periods of allometric growth. ^1^Allometric mammary growth in ruminants before the onset of puberty. Illustration of developmental changes and features is not to scale.

The mammary glands (MGs) produce the “perfect” food as milk. However, they must first assume their anatomical form—in this way the organ is unique, given most of its development occurs after birth. Not surprisingly, this development is tightly coordinated with a female’s developmental and reproductive states, as directed by the species-specific endocrine environment accompanying them (Hovey et al., 2002) (Figure 1). However, not all species undergo the same anatomical or morphological development, which remains an understudied area for applications ranging from lactation to cancer (Rowson et al., 2012). Finally, while there is a range of influences that can impact gland growth, none is as pronounced as the diverse and pleiotropic effect of a female’s nutritional status.

Our goal is to highlight the key anatomical similarities and differences in the MGs across species and during their postnatal development, with an emphasis on the endocrine factors regulating this development. At the same time, we address the role for nutrition during this process and highlight opportunities for further exploration. We anticipate these comparative revelations and the discussion of gaps in knowledge will propagate areas for future research as well as improved animal performance and human health.

## Overview of MG development

### Embryogenesis and MG positioning

During embryogenesis the milk “lines” form along the ventral body, thereby dictating symmetrical positioning of the future MGs. In a process of inductive signaling, the mesenchyme (as the future connective tissues of the gland) triggers epidermal cells at the outermost layer of the embryo to yield epithelial cells at locations of the future glands. These epithelial cells continue to cross-talk with the surrounding mesenchyme during sex- and species-specific development. By birth, there is an epithelial rudiment at the future site of the teat/nipple, in close and intimate association with a mixture of stromal cell types, ranging from adipocytes, fibroblasts and immune cells, to primordial cells of the vasculature and lymphatic systems (Hovey et al., 1999). Depending on the species, each gland may have multiple independent epithelial rudiments. The teat or nipple serves as the external apparatus for infant suckling and milk removal from the future epithelial structure (where a teat is defined as the external apparatus drained by a single duct/galactophore, whereas a nipple is drained by at least two of these). As an illustration of this cross-species variation, each MG in mice, cows, and goats has a single galactophore draining each teat, whereas each nipple in pigs externalizes two galactophores from independent epithelial structures, while the nipple of a human breast can have 8–20 galactophores (Russo et al., 2001; Rowson et al., 2012).

Developmental programming of the future subcutaneous mammary apparatus is diverse across species. For example, mice and rats have 5 and 6 pairs of MG positioned along the ventral midline, respectively, similar to the positioning in other litter-bearing species. In contrast, ruminants such as sheep, goats, and cattle have an inguinal collection of 2 or 4 glands forming a pendulous udder suspended from the pelvic floor, while elephants have a thoracic udder comprising 2 glands (Turner, 1952). The breasts of humans and nonhuman primates are positioned thoracically, while the 4 glands of macropod marsupials such as kangaroos and wallabies are positioned within the pouch. Glandular positioning is also genetically and epigenetically regulated; while some propose that MG number evolved proportional to the number of offspring, there is also a heritable genetic element to the positioning of teats/nipples, where selective breeding for this trait is commonplace in the pig industry (Felleki and Lundeheim, 2015). Furthermore, while symmetry is the basis for glandular positioning in all species, an uneven number of glands is not unusual, as is frequently the case in pigs. In this way, Alexander Graeme Bell undertook breeding studies in sheep that produced ewes having several more teats than their founders (1899). There is also an epigenetic component to gland positioning in that adults in species ranging from humans to livestock can present with supernumerary teats/nipples (Turner, 1952; Grossl, 2000; Oftedal and Dhouailly, 2013).

## Modeling postnatal MG development in rodents

The mouse has become a mainstay model for defining MG development, although its widespread use ignores the many differences that it holds relative to MGs in other species. Regardless, many of the fundamental processes of MG development are conserved, warranting a review of its MG development here.

**Figure 2.**
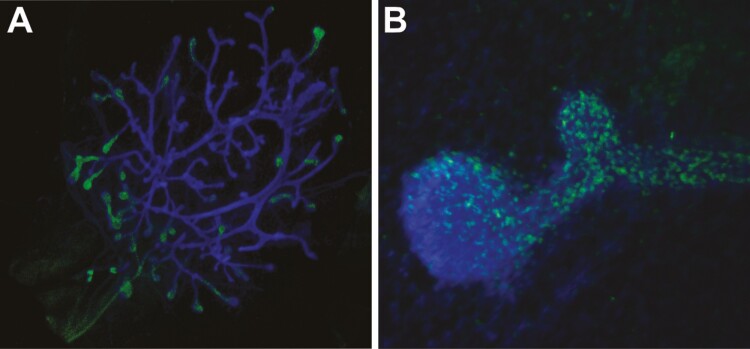
Fluorescent labeling of mitosis in terminal end buds in the mammary glands of ovariectomized mice after treatment with exogenous estrogen for 156 hours. Proliferating epithelial cells were detected by 5-ethynyl-2′-deoxyuridine (EdU) histochemistry (green) overlaid with DAPI (blue). A) Entire ductal network showing the abundance of EdU-positive cells in the terminal end buds. B) Higher magnification of EdU-positive terminal end buds.

At birth the simply-branched, canalized single epithelial rudiment is positioned above the teat and lies closely juxtaposed to the adipose-rich stromal microenvironment that will become its supporting matrix for the remainder of its development (Hovey and Aimo, 2010). Prior to puberty, the epithelium grows isometrically, namely at the same rate as the rest of the body (Hovey et al., 2002). With the onset of puberty, the gland enters an allometric growth phase when the ductal epithelium proliferates at a rate faster than the rest of the body. Morphologically, the ductal tips have enlarged monopodial termini, or terminal end buds (TEBs), that are filled with abundant mitotic epithelial cells arising from the pluripotent leading cap cells (Figure 2) (Paine and Lewis, 2017). Of course, one might expect that this concentrated zone of epithelial proliferation would amass a solid collection of epithelial cells, thereby precluding the formation of a canalized ductal system required for future drainage of milk to the teats. Instead, a proportion of newly formed epithelial cells almost immediately undergo apoptosis to clear a hollow luminal cavity within the center of the TEB (Paine and Lewis, 2017). The uniqueness of these TEB is even more pronounced when considering that the leading cap cells concurrently yield a unique population of contractile myoepithelial cells that arrange longitudinally on the basal edge around the subtending tubular ducts. Finally, a singular advancing TEB will bifurcate as the ducts advance, creating a branched ductal network that fills the mammary fat pad after several rounds of estrous cycling. Importantly, TEB never encroach closer than 0.25 mm on surrounding ducts, ensuring the ductal network retains an unrestricted yet volume-optimized ductal network, all as the result of local signaling and control mechanisms that are poorly understood (Hovey et al., 2002). Ductal elongation is complete once the mammary fat pad is completely filled by the ductal tree; in mice, the mammary fat pad is mostly composed of white adipocytes, and concurrently undergoes neovascularization and lymphangiogenesis as the ductal epithelium advances (Hovey and Aimo, 2010).

A range of inherent factors, besides the endocrine environment, influences development of the ductal network in mice, which informs our understanding of related processes in other species. For example, the rate and extent of ductal elongation and also branching morphogenesis in sexually-mature female mice is genetically-influenced, as highlighted by the range of phenotypes present in the MGs of various strains (Hadsell et al., 2015). In the same way, despite their similarity to mice, female rats develop a ductal network that is much more sympodially branched (Russo and Russo, 1978). The influence of genetics is also evident in male mice from different strains, where they may or may not survive androgen-induced destruction of the ductal rudiment in utero, or may develop a ductal network at various positions. Several environmental factors also determine the ductal phenotype. For example, the supportive extracellular environment of white adipose tissue cannot be replaced by any other tissue matrix (Hovey and Aimo, 2010), albeit that the ducts can (and do, in the case of the thoracic MGs) also grow into brown adipose tissue (Hovey and Aimo, 2010). Anatomic positioning of the glands also influences the progress and advancement of the ductal epithelium, where anterior glands in mice are smaller. Once the ductal network has filled the mammary fat pad, it resumes an isometric rate of growth, undergoing low levels of mitosis in its lateral branches with each recurrent estrous cycle.

While growth of the ductal network during puberty is certainly expansive, the amassing of epithelial cells in the gland during pregnancy is far greater (Tucker, 1987). At the morphological level, clusters of grape-like alveoli sprout from lateral tertiary branches scattered throughout the ductal network. Each alveolus contains ~100 epithelial cells, with each draining to its subtending duct via the hollow lateral branch on which it arose. Clusters of alveoli are arranged as lobules, and these are aggregated into lobes (Turner, 1952).

Alveologenesis is perhaps most pronounced, and critical, at the histomorphological level. Unlike their ductal neighbors, alveoli develop with a single layer of epithelial cells lining a hollow lumen (Brisken and Rajaram, 2006). On their basal side is a loose network of myoepithelial cells in a stellate arrangement, so as to realize milk ejection in response to oxytocin. The arrangement of epithelial cells into this discrete three-dimensional structure not only depends on the formation of lateral junctions between individual epithelial cells but also through their attachment to a mixture of basally-positioned extracellular matrix molecules (Brisken and Rajaram, 2006). As the lobulo-alveolar network assumes its arrangement, so too do the surrounding vascular and lymphatic systems. Proliferation and expansion of the alveolar tissue continues up until parturition, at which time there can also be concurrent cell division alongside the biochemical initiation of milk synthesis during lactogenesis (Tucker, 1987).

As mentioned above, the anatomy and morphological development of the MGs in mice is distinct from that in other species (Rowson et al., 2012). Our objective hereafter is not to provide an exhaustive account of mammary development across various species, but rather to highlight the key control points for each, and how this course of development differs from that in mice.

## Mammary development in pigs

Morphological progression of the mammary epithelium in sows more closely resembles that seen in the human breast (Horigan et al., 2009), placing its development somewhere between the simple ductal network in mice, and the more-dense and branched lobular growth in ruminants (Rowson et al., 2012). After birth, the ductal network in pigs expands via tubulo-lobular progression, with ductal elongation directed by TEB-like features. At the same time, there are branched lobular structures at the ends of ducts that can be defined as terminal ductal lobular unit (TDLU) structures; these TDLU have increasing complexity (TDLU1-4) that reflects the number of smaller ductules clustered on each TDLU. The development of these TDLU in pigs has been defined using the same scale as that used to characterize TDLU1-3 in humans (Horigan et al., 2009). The expanding ductal network also becomes interspersed with “intralobular” connective tissue, as occurs in the human breast (Hovey et al., 1999). As the ductal network grows into the surrounding white adipose tissue, its structures also become embedded in collagenous connective tissue that becomes the supporting “interlobular” stroma (Hovey et al., 1999; Horigan et al., 2009). Like in other species, the glands of sows undergo massive amounts of epithelial expansion during gestation, where the anterior glands are largest, while the posterior glands are smallest and least-developed.

## Mammary development in ruminants

The MGs of ruminants are unique by virtue of their arrangement as independent glands within the inguinally-positioned udder complex. The udder therefore comprises either 2 glandular halves in sheep and goats, or 4 glandular quarters in cows (Turner, 1952).

**Figure 3.**
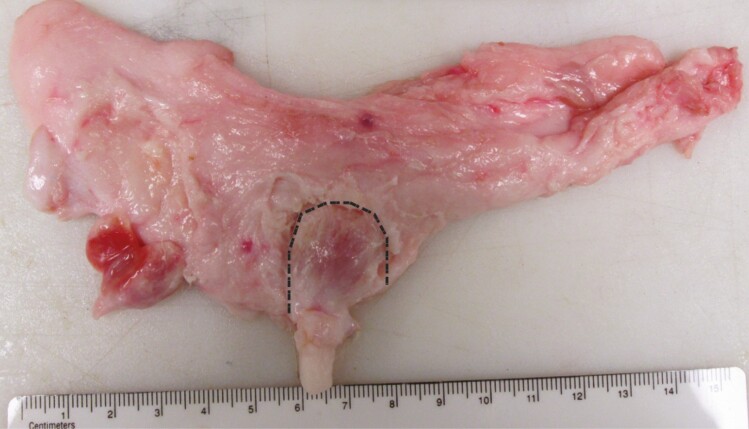
Cross section of the mammary gland from a 10-week old heifer calf fed a high plane of nutrition. The black dashed line circumscribes the parenchyma above the teat.

As an example, mammary growth in fetal heifers commences after the mammary bands appear around day 75 of gestation. Separate basal and luminal epithelial cells are subsequently evident in the mammary bud, which go on to yield the future myoepithelium, and secretory ducts and alveoli, respectively. By birth, the epithelial parenchyma is arranged as a rudimentary set of ducts at the base of the teat, with several histomorphological characteristics distinguishing it from that in rodents. First, there is no sexual dimorphism of the glands, so that both newborn male and female calves have mammary parenchyma above the teat, drained by a single galactophore. Second, the future gland cistern is distinguishable as an enlarged cavity juxtaposed to the teat (Capuco et al., 2002) (Figure 3). Third, a dense layer of interlobular stromal connective tissue envelops the branched epithelial parenchyma and separates it from adipocytes of the distal mammary fat pad. Finally, basally-positioned myoepithelial cells are not continuous around the epithelial parenchyma (Capuco et al., 2002). Similar developmental features and changes have also been described for the MGs of ewe lambs (Hovey et al., 2000).

**Figure 4.**
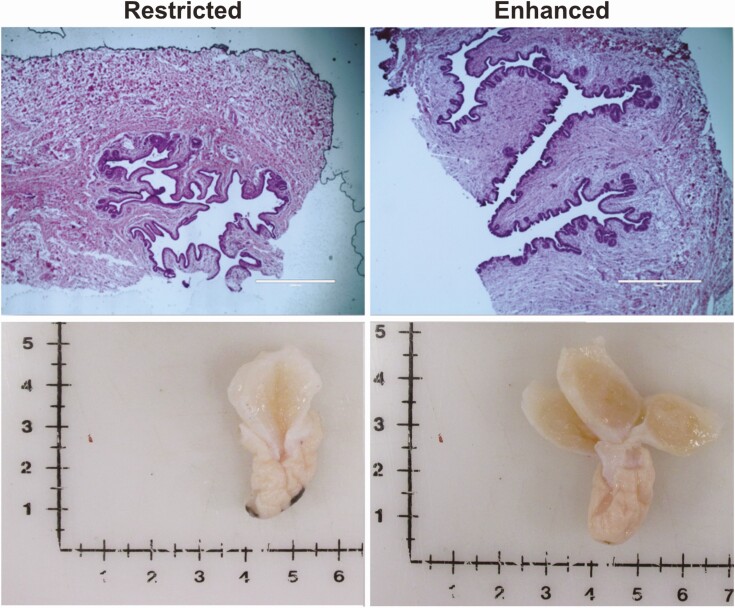
Mammary gland development in heifers necropsied at 8 weeks of age (at weaning) that were fed either a 20% crude protein, 20% fat milk replacer (restricted), or a milk replacer containing 28% crude protein and 25% fat (enhanced). Republished in part from Geiger et al., (2016a) J. Dairy Sci. 99:3995-4004 and Geiger et al., (2016b) J. Dairy Sci. 99:9, 7642-7653.

The typical female dairy calf is weaned around 2–3 months of age, when a traditionally-defined period of allometric growth was historically assumed to begin (Tucker, 1987). This period of mammary growth was long-considered to be an important determinant of the future lactational performance of heifers. However, subsequent studies have challenged this dogma, pointing to the first 2–3 months of life as being a period of active mammary growth. Indeed, the MG parenchyma increases in size by greater than 60-fold during this time, while body weight only doubles (Capuco and Akers, 2010) (Figure 4).

The developing epithelial parenchyma assumes a histomorphology comprised of TDLU-like epithelial structures that are sympodially-branched (Capuco et al., 2002). Unlike in mice, the parenchyma advances into the surrounding adipose-rich mammary fat pad *en bloc*. In ruminants, the adipose environment of the mammary fat pad is also invested with a mesh-weave of connective tissue veins that become the collagenous inter- and intralobular fibrils dispersed throughout the parenchyma (Hovey et al., 1999). Previously we proposed that these connective tissue elements likely provide lifelong support for the pendulous udder (Hovey et al., 1999).

## Breast development in humans

Our objective here is to highlight similarities and distinctions between the MG of humans and other species. A key feature of the human breast is the 8–20 independent ducts/galactophores draining each nipple that are present in both males and females (Russo et al., 2001). Although rudimentary, the mammary epithelium of newborn humans can demonstrate precocious milk secretion following stimulation by maternal hormones, a phenomenon referred to as “witches milk”. With the onset of puberty, the 8–20 ductal networks then undergo a complex histomorphological progression, where ducts advance into the surrounding adipose tissue as led by enlarged ductal termini, seemingly reminiscent of TEB in rodents (Russo et al., 2001). However, the precise histological properties of these structures in humans remain poorly characterized.

The ductal network also assumes a complex morphology, having branched and lobulated TDLU structures at the ductal termini. Notably, these TDLU develop morphologically over time and in response to hormonal stimulation—the most simple TDLU (TDLU-1) contains an average of approximately 11 terminal branches, or “ductules”, and are present in the earliest stages of postnatal breast development. These TDLU-1 can then assume a more complex and branched phenotype as they progress developmentally, giving rise to TDLU-2 and -3 during sexual maturation. The TDLU-2 and -3 have been characterized as having an average of 47 and 80 ductules, respectively (Russo et al., 2001). The morphological progression of these TDLU is likely realized by sympodial branching of the terminal ductules. The most developed lobulated structures, TDLU-4, then develop during gestation as a function of further lobulation and expansion of discrete alveolar structures.

Like in pigs and ruminants, each ductal network in the human breast undergoes histomorphological development within a specialized stroma of connective tissue. Specifically, each TDLU has a distinctive “intralobular” connective tissue matrix, while the entire advancing TDLU becomes enveloped with “interlobular” collagenous fibrils as it invades the surrounding adipose tissue (Hovey and Aimo, 2010). In turn, these inter- and intralobular connective tissues associate with Cooper’s ligaments that provide additional physical support to the suspended breast via connection to the clavipectoral fascia.

## Endocrine regulation of mammary development

### Cell types within the MGs as endocrine targets

Numerous hormones, cell-surface receptors and intracellular signaling cascades mediate the actions of hormones acting on the MGs that have been reviewed extensively elsewhere (Hovey et al., 2002). That said, a key point is that hormonal effects that are manifest as specific phenotypic responses by subpopulations of epithelial cells in the growing MGs are infrequently the result of direct targeting of that same population.

One example is the mediatory role of various nonepithelial cell types within the mammary stroma, whether they be adipocytes, connective tissue fibroblasts, various immune cell types, or the vasculature and lymphatics, where each of these cell populations has been implicated as a local mediator of systemic endocrine signals (Hovey et al., 1999). Specifically, each of these cell types can synthesize and secrete positive and negative growth regulators, including the epidermal growth factors, the fibroblast growth factors, and wingless-related integration site factors (Wnts). On this point, these local regulatory molecules are not only limited to protein growth factors but also include growth-modulating fatty acids from adjacent adipocytes (Hovey and Aimo, 2010), or their metabolites including leukotrienes and prostaglandins. In a similar vein, local signaling relies on the generation of several different amino acid metabolites to yield local growth regulators such as histamine, nitric oxide, and serotonin. All these examples highlight how the dynamic regulation of MG growth and function is at the intersection of endocrinology and nutrition.

While this multifactorial strategy underlying hormonal regulation quickly becomes complex, these additional layers of modulation are likely essential for reasons ranging from a) the ability to confer a tissue-specific response to an otherwise systemic hormone, b) to provide additional sensing of the overall physiological state of the female, such as for her plane of nutrition, c) the opportunity to target subpopulations of cells, or d) to converge several of these pathways to integrate and build additional layers of targeted regulation of the mammary epithelium.

Within a gland, not all epithelial cells are identical, or respond in the same way to different hormones. Furthermore, signaling between different heterogeneous cells in the epithelium is critical for mediating the actions of hormones such as estrogen (E), progesterone (P), and prolactin (PRL). For example, in the case of ovarian hormone signaling, several lines of evidence suggest that steroid-sensing epithelial cells send paracrine cues to neighboring cells that then divide (Capuco et al., 2002; Clarke, 2006). In the same way, there is heterogeneous distribution of P and PRL receptors (PR and PRLR) in luminal epithelial cells (Hovey et al., 2001), in keeping with their ability to sense these hormones and then activate paracrine growth factor signaling via molecules such as insulin-like growth facor (IGF)-2 and Wnts (Brisken et al., 2000; Hovey et al., 2003). The full extent of how local cross-talk between subsets of individual epithelial cells occurs after hormone action will remain challenging to unravel given the dynamic and cell-by-cell responses involved.

Likewise, several lines of attention have been given to the myoepithelial/basal population of epithelial cells that can also regulate paracrine signaling to the adjacent luminal epithelium. For example, the myoepithelium was hypothesized to modulate the response of the epithelium to the actions of various growth factors (Ballagh et al., 2008). Similarly, decreased epithelial proliferation in ovariectomized calves was hypothesized as being due to altered myoepithelial cell morphology (Ballagh et al., 2008).

Taken together, clearly there is a multitude of pathways and regulatory steps that can mediate the actions of a wide range of endocrine hormones at play during MG development. While roles for many of these candidate mechanisms have been resolved in mouse models, the real test becomes whether these pathways are also functional in glands having different morphologies and growth trajectories, whether that be for humans or livestock.

## Ovarian regulation

The ovaries and their hormones, E and P, are essential for postnatal ductal development, as highlighted by the fact that ovariectomy or disrupted ovarian function in nulliparous females blocks allometric growth of the ductal network in rodents, ruminants, pigs, and humans (Turner, 1952; Hovey et al., 2002). Extending this knowledge a step further, the effects of ovariectomy can also serve to reduce mammary tumorigenesis in humans and companion animals. Interestingly, ewe lambs appear to be unique in this regard given that prepubertal ovariectomy did not block allometric growth of the glands (Ellis et al., 1998). This peripubertal phase of allometric growth is attributable to the actions of E, given that E-replacement can rescue ovariectomy-suppressed ductal growth in all the aforementioned species (Hovey et al., 2002; Rowson et al., 2012).

Broadly speaking, subsequent branching and lobular development of the mammary epithelium during recurrent estrous cycles depends on the combined and coordinated responses to E and P. In this way, E induces epithelial expression of the PR, which is a conserved response across species (Hovey et al., 2002). Epithelial PR then subsequently facilitate P-induced proliferation within a subpopulation of the mammary epithelium to realize branching morphogenesis and alveologenesis. While epithelial cells are responsive to P in vitro, there are several indications that this response in vivo also involves the local synthesis of a range of paracrine growth regulators derived from the stroma, or from adjacent, nondividing but PR-positive, epithelial cells (Capuco et al., 2002; Clarke, 2006). As noted below, the response to P is also modulated by pituitary-derived PRL and/or growth hormone (GH).

## Growth hormone and insulin-like growth factor-1 (IGF-1)

While the ovaries are essential for MG development, their effects also require a functional pituitary gland as the source of GH (Lyons et al., 1958). From an elegant series of experiments in rodents, it became clear that during this combined endocrine response, GH acts on adipocytes of the mammary fat pad to provide a local source of IGF-1, which then acts as a paracrine mitogen to stimulate epithelial proliferation (Kleinberg and Ruan, 2008). In rodents at least, this paracrine IGF-1 then induces TEB formation, followed by ductal elongation (Kleinberg and Ruan, 2008). In ruminants it is likely that the situation is similar, given that the MG stroma synthesizes IGF-1 at the same time there is the onset of allometric growth prior to puberty (Hovey et al., 1998; Akers et al., 2005). Such a mechanism is consistent with the fact that both GH and local IGF-1 can stimulate growth of the mammary epithelium of heifers in vivo (Akers et al., 2005). Not surprisingly, these effects of IGF-1 happen in concert with the actions of E that concurrently enhances GH-induced synthesis of IGF-1 by the stroma (Akers et al., 2005; Kleinberg and Ruan, 2008); in turn, GH induces the expression of stromal E receptors (ER) that presumably further amplifies this local growth-stimulating mechanism (Kleinberg and Ruan, 2008).

## Prolactin

Pituitary-derived PRL not only fulfils its well-documented and essential function during established lactation, but also plays a critical role during gland growth. While a role for PRL is less compelling during ductal growth, several insights raise the possibility that it does contribute during this period. The mammary epithelium is responsive to PRL during this period in vivo (Hovey et al., 2001), although ductal growth in mice still occurs following deletion of the PRLR. One unresolved question is whether PRL plays a greater role during this phase in species that undergo a more complex branching morphogenesis, including humans and ruminants. Such a suggestion gains support from the demonstration that only a subset of epithelial cells in the mammary ducts expresses PRLR (Hovey et al., 2001); as highlighted below, any actions of PRL during pregestational growth may depend on its cooperativity with ovarian hormones such as either E or P.

Transitioning of the gland to gestational growth involves an essential role for PRL, in keeping with the finding that the absence of either PRL or the PRLR in mice prevents lobuloalveolar development during gestation. In the same way, suppression of PRL during pregnancy blocks mammary development in sows (Farmer and Petitclerc, 2003). Of course, PRL not only promotes extensive growth of the alveolar epithelium during gestation but also plays a major role in coordinating the epigenetic and biochemical changes required for lactogenic differentiation that have been reviewed extensively elsewhere. Finally, it is worth highlighting that as occurs during pregestational growth, the secretion and actions of PRL are sensitive to ovarian activity, where PRL secretion by the pituitary increases during gestation and is increased by rising E levels.

## Placental lactogen

While there is undoubtedly a role for placenta-derived placental lactogen, which has PRL and GH-like activity, during gestation-associated MG development, its effects remain grossly understudied. Several key points are worth mentioning. First, species vary in their synthesis of placental lactogen, where its contribution to MG growth is likely species-dependent (Forsyth, 1994). Second, cross-reactivity between placental lactogen and the various lactogenic receptors (GHR and PRLR) is without doubt, which complicates any interpretation of how crucial placental lactogen is for mammary growth. This cross-reactivity between ligands and receptors also varies by species (Forsyth, 1994). Last, the actual role for placental lactogen during different stages of MG growth and early differentiation is often challenging to distinguish given the concurrent roles for PRL and GH. Needless to say, there are several compelling examples whereby placental lactogen clearly stimulates MG development during gestation. Highlighting this point, circulating levels of placental lactogen are proportional to the size of the placenta and the number of offspring (Thordarson and Talamantes, 1987), which is subsequently evident as a proportional increase in MG growth and milk production (Thordarson and Talamantes, 1987).

## The potential of hormone interactions

As outlined above, there have been many important insights stemming from studies focused on defining how individual hormones direct MG growth. Having said that, no hormones act on their target tissues in isolation, and the developing MGs are no exception. Very few studies have explored these interrelationships, perhaps because of the complex and multifactorial basis for such questions, and/or because of simple economics. Here, we briefly review some of the gaps and questions that exist in this space.

There is strong consensus that the mainstay endocrine regulators of ductal growth are E and GH, as mediated by local IGF-1 synthesis (Akers et al., 2005; Kleinberg and Ruan, 2008). However, additional factors likely contribute during this stage of development. For example, Kleinberg and colleagues found that the actions of P also interacted with IGF-1 to stimulate ductal growth in rats (Ruan et al., 2005), although the extent to which this unique combination contributes to normal development has not been investigated. Perhaps in a similar way, local regulators such as amphiregulin (that is E-induced) not only act independently but also engage the signaling for other molecules including IGF-1.

In a similar fashion, E+P is frequently considered a mainstay hormone combination that promotes branching morphogenesis to realize a phenotype that differs somewhat depending on the species (Hovey et al., 2002; Horigan et al., 2009). We wish to highlight that these responses to E+P almost always likely involve an unaddressed cooperativity with endogenous pituitary hormones, especially PRL, that is also induced by exogenous E. In fact, any endocrine experiments studying the effects of individual hormones or their combinations on the MGs should be performed in either ovary- and pituitary-ablated animals, genetic models blocking specific hormone receptor pathways, or in the presence of chemical antagonists (Horigan et al., 2009). These approaches can all reveal important additional information including unique hormone combinations that are likely required for species-specific development. For example, P+PRL stimulates considerable epithelial proliferation and branching morphogenesis in mice without any requirement for E (Hovey et al., 2001). Interestingly, this notion that P cooperates with a pituitary hormone is reminiscent of the finding that P can induce the local synthesis of GH in the MGs of female dogs. A different scenario exists for the endocrine combinations that direct TDLU development in the MGs of pigs, where the combination of E+PRL synergistically induced epithelial division and TDLU progression (Horigan et al., 2009), whereas P+PRL (that had a pronounced impact on the MGs of mice (Hovey et al., 2001)) was without effect.

## Nutritional regulation of MG development

While the endocrine environment is the primary regulator of mammary development, nutrition can also impact the process in several ways. This impact should perhaps not be surprising—the gland develops during postnatal life when the female’s nutritional state can, and will, change appreciably; it develops in intimate association with nutrient-sensitive adipose tissue; and it is inherently preparing for lactation when it becomes one of the most nutrient-sensitive and -demanding organs of the body. In the following section, we touch on some notable examples of how different nutritional considerations can impact the course of MG development.

## Effects of plane of nutrition and its temporality

There remains a continued interest in how caloric intake can impact MG development, ranging from how it affects future milk production potential to how it impacts breast cancer risk. In mice, reduced caloric intake impaired mammary development, whereas excess caloric intake increased growth and subsequent branching and alveolar development (Engelman et al., 1993). Similarly, in species such as pigs (unpublished observations) and in humans, excess energy intake during ductal elongation impairs development, and may reduce a woman’s risk for developing breast cancer later in life.

In ruminant animals, especially dairy heifers, excess energy intake and rapid growth during early development has long been linked to impaired allometric growth of the gland, along with pronounced negative impacts on future milk production (Sejrsen et al., 2000). Within this phenotype there are clear relationships between the circulating levels of GH and IGF-1 and mammary growth during this period and its response to plane of nutrition. However, increased energy intake also increases somatic growth, maturation of the hypothalamic/pituitary/gonadal axis, leading to the earlier onset of isometric mammary growth following puberty. As a result, when chronological versus physiological age was accounted for, the excess nutrient intake between weaning and the onset of puberty was not detrimental (Meyer et al., 2006). The gland seems to be very nutrient-responsive during this period of allometric growth, where its growth is positively correlated to greater nutrient intake prior to weaning (Geiger et al., 2017) (Figure 4). These and related insights revealed that in heifers, allometric growth of the glands likely begins around birth, and have led to revised strategies for maximizing heifer growth and future milk production potential, particularly before weaning. Dairy producers now frequently feed upward-revised levels of energy to preweaning heifers, given that every kilogram of average daily gain by calves is projected to increase first-lactation milk yield by 1550 kg (Soberon and Van Amburgh, 2013).

Excess nutrition appears to have less of an impact on MG growth and development in post-pubertal heifers followed into gestation. There is clear recognition that overfeeding sows during gestation can impair their future milk production, not only because of negative impacts on gland development but also due to altered physiological adaptation for lactation. These considerations are equally evident in humans, where obesity and metabolic dysregulation during pregnancy are often associated with breastfeeding struggles, with several lines of evidence pointing to impaired glandular development as being one of the root causes for this outcome.

## Irregular rates of gain

Stair-step or compensatory feeding programs that expose animals to alternating periods of restricted or excess feeding have been used to minimize somatic growth during the restrictive phase, and to accelerate it during the excess stage. Ford and Park assessed the effect of a compensatory feeding program in heifers using a 3/2, 4/3, 4/2 month schedule starting at 6 months of age, where the first 3 months was a restricted phase, followed by a 2 month excess stage, with subsequent alternating restrictive and excess phases (Ford and Park, 2001). This feeding program increased first and second lactation milk yields by 21% and 15%, respectively. The authors hypothesized these results may be due to compensatory mammary epithelial cell proliferation, a phenomenon that has also been observed in mammary tissue from rats during compensatory growth phases during pregnancy and early lactation (Kim et al.,1998). In a similar way, feeding heifers on a compensatory stair-step regimen increased the content of DNA, RNA, and protein, and the RNA:DNA and protein:DNA ratios, in their mammary glands by mid- to late-pregnancy.

## Growth regulation by fatty acids

Unsaturated fatty acids having one or more double bonds in their carbon chain, such as linoleic acid, have long been implicated in the regulation of mammary development, where supplemental unsaturated fatty acids have consistently been found to stimulate epithelial growth in vitro. Indeed, supplementing the diet of female mice with various unsaturated fatty acids increased mammary growth (Welsch and O’Connor, 1989). In contrast, diets supplemented with saturated fatty acids lacking double bonds led to impaired mammary development (Welsch and O’Connor, 1989). In a similar way, feeding ewe lambs a diet supplemented with protected polyunsaturated fats increased the amount of parenchyma in the MGs (McFadden et al., 1990), although heifers fed diets rich in soybean oil had no change in their body weight or mass of either the mammary parenchyma or fat pad, despite having an increased concentration of 18:1 in the plasma (Thibault et al., 2003). A notable outcome from that study was that while the treatments ended at 6 months of age, heifers fed the high oil diet retained a mammary fat pad that weighed 10% less than in control animals 6 months later.

**Figure 5.**
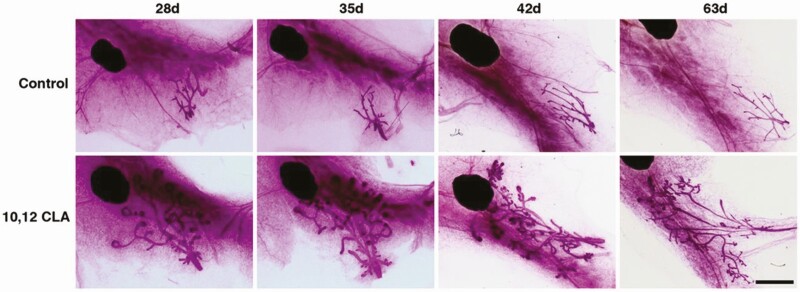
Effect of *trans*-10, cis-12 conjugated linoleic acid (CLA) on the mammary glands of ovariectomized mice. Representative mammary gland whole mounts from ovariectomized Balb/cJ mice fed either a control (top row) or 1% 10, 12 CLA diet (bottom row) from 22d, then euthanized at 28, 35, 42, or 63d. Scale bar is 2 mm. Republished from Berryhill et al., (2012) Proceedings of the National Academy of Sciences, 109:40, 16294-16299.

While various unsaturated fatty acids can certainly influence various aspects of mammary growth, it is their unique isomers that provide intriguing insights into the regulation of glandular development by lipids. Among these, *trans*-10, cis-12 conjugated linoleic acid appears to exert some of the most pronounced effects. Specifically, when a diet containing 1% 10,12 conjugated linoleic acid was fed to ovariectomized prepubertal mice, the glandular epithelium initiated allometric growth including the formation of TEB, even despite the absence of any E stimulation (Berryhill et al., 2012) (Figure 5). In contrast, other *trans* fatty acids were without any pronounced effect in this way. A common mechanistic thread underlying these effects may reflect 10,12 conjugated linoleic acid impacting the stromal microenvironment, given that dietary 10,12 conjugated linoleic acid not only induces lipolysis in mammary adipocytes alongside local inflammation in the vicinity of growth-stimulated mammary epithelium, but it also upregulated the local presence of stimulatory paracrine factors such as IGF-1 and epidermal like growth factor-like ligands (Berryhill et al., 2012, 2017). In contrast, feeding *trans* fatty acids fed to calves apparently has a less pronounced effect on parenchymal development.

## Conclusion: not all MGs develop similarly

Across species, the MGs are as diverse in their development as they are in their response to the factors regulating it. From a comparative standpoint, many questions remain about how the histomorphological phenotype directs a range of phenotypic outcomes that can have wide-ranging impacts from milk production potential to cancer risk. We contend there are many unique opportunities to determine the regulation of these processes from a cross-species perspective. The significance of the many hormone interactions that drive glandular development during puberty and gestation across a range of species also cannot be understated. And while advances have been made for better nutritional management of dairy animals to enhance milk production, our ability to nutritionally modulate development for lactational success, or for a reduction in breast cancer risk, remains lacking.

## About the Author

**Dr Adam Geiger** was raised on a dairy farm in Wisconsin. He received his B.S. from the University of Wisconsin-Madison in Dairy Science. Upon graduation, he completed a M.S. degree at Mississippi State University in Ruminant Nutrition. Adam then received his Ph.D. from Virginia Tech in Lactation Physiology. His dissertation is a series of work dedicated to understanding the relationship between nutrition and the mammary gland and understanding how both interact with various hormonal axis to improve milk production. Adam then spent time with a large animal nutrition company before starting with Zinpro as a Research Nutritionist in 2017. Adam has been an invited speaker and/or conducted technical service visits in 46 states and 58 countries throughout the world. Now, Adam is a business development manager for Zinpro and continues to provide technical service globally. Adam currently resides in Madison, Georgia (USA) with his wife, Abby, and their 2 kids, Abram (8) and Adelyn (5).

**Professor Russ Hovey** received a Bachelors of Agricultural Science, with a major in Animal Science and First Class Honors, from the University of Queensland, Australia. He earned his PhD from Massey University, New Zealand while studying at the Ruakura Research Center and working on the role of the mammary fat pad during mammary development. He subsequently undertook postdoctoral at the National Cancer Institute in Bethesda, Maryland where he worked on the hormonal regulation of mammary gland development and breast cancer. He began his academic career in the Department of Animal Science at the University of Vermont, then relocated to the Department of Animal Science at UC Davis, where he teaches the Introductory Animal Science course and a senior-level course in Lactation Physiology. He also is the outgoing Editor in Chief for the Journal of Mammary Gland Biology and Neoplasia, and is co-Chair for the 2023 Gordon Research Conference on Mammary Gland Biology.
